# Tropical dry woodland loss in India since 1880 and its relation to current megafauna distributions

**DOI:** 10.1002/eap.70054

**Published:** 2025-07-07

**Authors:** Tamanna Kalam, Matthias Baumann, Florian Pötzschner, C. Sudhakar Reddy, Arash Ghoddousi, Parth Sarathi Roy, Tobias Kuemmerle

**Affiliations:** ^1^ Geography Department Humboldt‐Universität zu Berlin Berlin Germany; ^2^ Forest Biodiversity and Ecology Division, National Remote Sensing Centre Indian Space Research Organisation Balanagar Hyderabad India; ^3^ Wildlife Ecology and Conservation Group Wageningen University and Research Wageningen the Netherlands; ^4^ World Resources Institute Mumbai India; ^5^ Integrative Research Institute on Transformations of Human‐Environment Systems (IRI THESys) Humboldt‐Universität zu Berlin Berlin Germany

**Keywords:** archetype analysis, biodiversity, deforestation, forest transition, grasslands, large mammals, savannas

## Abstract

Tropical dry woodlands provide ecosystem services to hundreds of millions of people and support high biodiversity. Despite their importance, many dry woodlands are under high and rising human pressure, including in India, where they provide essential habitat for iconic megafauna. However, there are notable gaps in our understanding of long‐term changes in dry woodlands and how they relate to the present‐day distribution of megafauna. Here, we reconstructed tropical dry woodland change since the 19th century, identified archetypes of change, and explored their relationship with current megafauna distributions. More specifically, we compared the reliability of existing satellite‐based woodland maps and integrated them into an ensemble map of contemporary dry woodland cover in India. This allowed us to derive recent changes in dry woodlands since 1995 and, by integrating them with historical maps, long‐term changes since 1880. Finally, we used non‐parametric spatial clustering to detect typical patterns of long‐term woodland change and compared these to the current distribution of 14 megafauna species. These analyses yielded four major insights. First, we show a massive historical loss of dry woodland cover in India since the 19th century, with over 22 Mha (equaling 65% of dry woodlands) lost, underscoring the threatened nature of these ecosystems. Second, we identified six archetypes of woodland change, three characterized by different levels of continuous woodland decline and three showing a forest transition pattern of historical decline, stability, and subsequent recovery. This highlights the regional variations in woodland dynamics across India's dry woodlands. Third, we found a strong and positive link between current megafauna distribution and high woodland cover, especially for threatened species (*r* = 0.43, *p* < 0.05), regardless of woodland histories, pointing to the importance of maintaining larger tracts of dry woodlands for safeguarding megafauna and for megafauna restoration potential where woodlands are recovering. Finally, we show that Indian dry woodlands are still undergoing widespread losses of 6.5 Mha since 1995, and pressure on them has been increasing recently. Therefore, better protection and monitoring of dry woodlands is urgently needed, and our analyses can provide a basis for context‐specific land‐use and conservation planning.

## INTRODUCTION

Tropical dry woodlands, including tropical and subtropical dry forests, savannas, and shrublands, cover over a fifth of the global terrestrial surface (Dinerstein et al., 2017; Olson et al., 2001). They shelter 25% of the world's human population (Schröder et al., 2021) and provide a myriad of ecosystem services, including carbon storage (Portillo‐Quintero et al., 2015), food, firewood, and charcoal, as well as many non‐timber forest products (Djoudi et al., 2015; Schröder et al., 2021). However, despite their socio‐ecological importance, only a small proportion of dry woodlands are protected globally. For example, only 4% of Myanmar's dry woodlands (Songer et al., 2009), 4% of Brazil (Espírito‐Santo et al., 2008), or less than 5% of Latin America's dry woodlands (Portillo‐Quintero & Sánchez‐Azofeifa, 2010) are protected. Many dry woodlands have remained under the radar of research and conservation efforts owing to a bias toward the more charismatic rainforests (Santos et al., 2011; Siyum, 2020). Further, even among dry woodland regions, there is a geographical bias, with more effort focused on dry woodlands in Latin America and much less focus on African and Asian dry woodlands (Schröder et al., 2021).

The neglected nature of dry woodlands is worrying, as many dry woodlands globally are under high and rising human pressure. To date, nearly half of all tropical dry woodlands have been converted (Hoekstra et al., 2005), primarily because of their suitability for different forms of agriculture (Sanchez‐Azofeifa et al., 2005). For instance, producing commodity crops, such as soybeans, has led to rampant deforestation in the Dry Chaco, Cerrado, and Chiquitania regions of South America, turning them into deforestation hotspots (Buchadas et al., 2022). Similarly, the expansion of commodity crops such as oil palm, soy, maize, and cassava has led to the widespread conversion of dry woodlands in Sub‐Saharan Africa (Ordway et al., 2017). Even in Southeast Asia, agricultural conversion, primarily for sugar cane, drove dry woodland loss (Songer et al., 2009). Deforestation of tropical dry woodlands leads to globally relevant carbon emissions, erosion of ecosystem services, displacement of smallholders, and defaunation (Polaina et al., 2018; Ripple et al., 2015). Understanding recent and long‐term changes in these ecosystems is, therefore, vital.

Tropical dry woodlands are India's most widely distributed forest type and provide almost all of India's fuelwood and fodder supply (Singh & Singh, 2011). However, comparatively little is known about how they have changed historically. Understanding historical land‐use patterns is essential as the legacies of past forest use, deforestation, and recovery can be long‐lasting (Foster et al., 2003; Munteanu et al., 2015). India has a long history of exploiting dry woodland resources, especially during the colonial era when the British rampantly cleared dry woodlands to meet timber demands (e.g., for railway expansion and shipbuilding) and for expanding agriculture (Bhojvaid et al., 2016). Several efforts have been made to understand the long‐term changes in dry woodland cover in India. Yet, many of these are focused on recent changes and are limited in space, such as focusing on central India (Reddy et al., 2015) or the Eastern Ghats (Ramachandran et al., 2018). Other studies have assessed woodland change over long periods but using very coarse‐scale and simulated data (Moulds et al., 2018; Tian et al., 2014). We know of only one higher resolution reconstruction of forest change using topographic maps going back to 1880, but this assessment did not include recent changes (Reddy et al., 2017). Satellite‐based analyses have provided detailed characterizations of woodland cover (e.g., from 1985 to 2005; Roy et al., 2015), but they also do not cover the most recent periods. Finally, global forest cover maps have recently become available (Hansen et al., 2013), but their accuracy is variable, overrepresenting woodland cover in some countries and underrepresenting it in others (Tropek et al., 2014) or detecting high rates of deforestation in non‐forest cover classes (Bellot et al., 2017). Overall, despite the large area covered by tropical dry woodlands in India, a reliable, up‐to‐date map of these woodlands, as well as a robust assessment of recent and long‐term dynamics in them, is missing.

This is unfortunate because tropical dry woodlands harbor high biodiversity, particularly of large animals (hereafter: megafauna; species >20 kg), as they provide more palatable food for large herbivores than rainforests and thus also host a higher diversity of large carnivores (Fernando & Leimgruber, 2011). Megafauna are of conservation concern, as they are often vulnerable to habitat loss and direct exploitation, and they also occupy critical roles in food webs and influence the functioning of ecosystems in important ways. India stands out globally for maintaining exceptional diversity and abundance of megafauna. For example, India is a stronghold for 100% of the global Asiatic lion population (*Panthera leo persica*) (Singh & Gibson, 2011), over 70% of the global tiger (*Panthera tigris*) population (Jhala et al., 2014), and over 60% of the global Asian elephant (*Elephas maximus*) population (Baskaran et al., 2011). India's dry woodlands also support high densities of ungulates, such as chital (*Axis axis*) and sambar (*Rusa unicolor*) (Bagchi et al., 2004) and large herbivores such as gaur (*Bos gaurus*) (Choudhury, 2002). Although contemporary megafauna richness and abundance in India's dry woodlands are remarkable, it is important to recognize that megafauna distributions and populations have likely been severely decimated for centuries by the joint effects of land‐use change and overhunting (Karanth et al., 2010). Likewise, human pressure on dry woodlands has been rising in some areas (Agarwala et al., 2016; Baldi & Jobbágy, 2012), including for megafauna. For instance, tigers in India are disappearing faster in dry woodlands than in other habitats (Chundawat et al., 2016). Whether the current distribution of megafauna is related to stability and persistence in tropical dry woodlands, or instead, megafauna have persisted despite historical land‐use change and dry woodland loss is important to understand to help devise conservation strategies targeted at megafauna protection and coexistence with people.

We sought to assess the current distribution and long‐term changes in India's tropical dry woodlands by generating an ensemble map of dry woodland change from satellite‐based and historical maps covering a period of 140 years. We then use archetype analyses (Eisenack et al., 2021; Oberlack et al., 2023; Sietz et al., 2019) to identify major patterns of change and further relate these patterns to contemporary megafauna distributions. Specifically, we asked:

1. What are long‐term (since 1880) and recent (since 1995) changes in Indian dry woodlands?
2. What are the major patterns of dry woodland change in India since 1880?
3. How do these patterns relate to contemporary megafauna distribution?

## STUDY AREA

For our study, we consider those areas as tropical dry woodlands that fall within the biomes of (1) tropical and subtropical dry broadleaf forests or (2) desert and xeric shrublands according to the updated classification by Dinerstein et al. (2017), originally by Olson et al. (2001). This translates to what Champion and Seth (1968) define as Indian tropical dry forests, including (1) tropical dry evergreen forests, (2) tropical dry deciduous forests, and (3) tropical thorn forests. Thus, we use a broad definition of tropical dry woodlands, encompassing both dense and open woodlands and areas where tree or shrub cover is the dominant woody cover. This acknowledges that transitions between these vegetation types are often gradual and spatially diffuse, making the delineation of hard boundaries artificial. Tropical dry woodlands in India occur in areas with mean annual temperature between 18 and 35°C and annual rainfall ranging from 900 to 1500 mm (Champion & Seth, 1968). Geographically, dry woodlands are distributed from Tamil Nadu in southern India to Punjab in the north and from Gujarat to West Bengal in the east–west extent. This includes eight ecoregions: Aravalli, Khathiar‐Gir, Narmada Valley, Central Deccan Plateau, Chota Nagpur, Deccan Thorn, South Deccan Plateau, and East Deccan Plateau. Indian dry woodlands have lower species richness compared to rainforests; however, they still harbor many endemic and rare species of conservation concern (Singh & Chaturvedi, 2017).

Tropical dry woodlands have been under high pressure historically. During colonial times, dry woodland tree species such as teak (*Tectona grandis*) and sal (*Shorea robusta*) were intensively extracted for shipbuilding and railway sleeper beds (Bhojvaid et al., 2016; Gadgil, 1990). Later, during the Green Revolution, modern agricultural technologies transformed India's cropping systems, boosted farm production, and increased land under cultivation, leading to marked losses in dry woodland areas (Sannigrahi et al., 2021). In recent decades, infrastructure projects, particularly for the building of dams and canals, progressively increased cropland areas in many dry woodland regions (e.g., central India) (Roy et al., 2015). Other recent notable threats to dry woodlands are changes in fire regimes (Schmerbeck, 2011) and mining projects (Jayakumar & Arockiasamy, 2003). In response to these threats, several government policies, such as the Forest Conservation Act (1980) and the National Forest Policy of India (1952), have been enacted to lower deforestation, along with nationwide afforestation programs that have promoted the recovery of secondary forests (Bhat et al., 2001).

## METHODS

**FIGURE 1:**
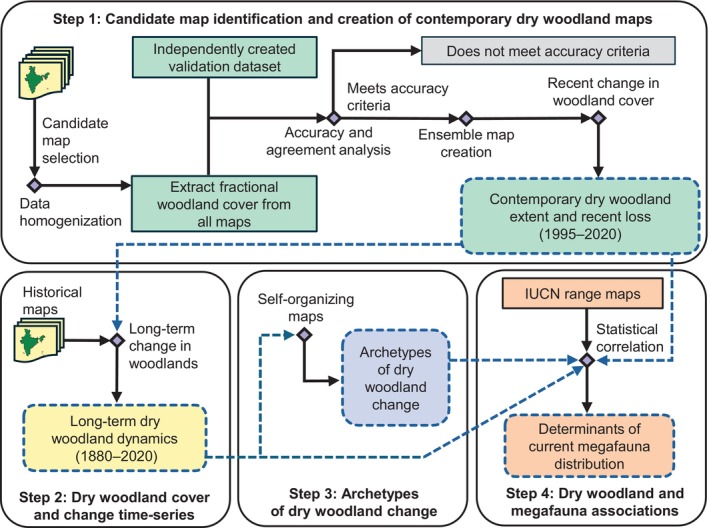
Overview of the analysis to (1) create contemporary extent and changes within dry woodlands by identifying candidate woodland maps using accuracy‐based measures, (2) recreate long‐term dry woodland change dynamics, (3) determine the major patterns of dry woodland change over time, and (4) determine the associations between contemporary and historic dry woodland cover and change, major patterns of woodland change, and current megafauna distribution.

Our analysis involved four main steps (Figure 1). In step one, we identified candidate dry woodland maps and created a contemporary dry woodland cover map for 2010 and 2020. In step two, we recreated the full time series of dry woodland cover and change maps going back to 1880. In step three, we clustered our time series to determine the major patterns of dry woodland change in Indian dry woodlands, which we refer to as archetypes of dry woodland change. Finally, in step four, we related dry woodland cover change and our archetypes to the ranges of 14 megafauna species to understand the relationship between dry woodland change and contemporary megafauna distribution.

### Contemporary extent and recent changes in India's tropical dry woodlands

We identified seven datasets providing dry woodland cover for our entire study region (Table 1) for ca. 2010 and 2020. We created fractional woodland cover maps for each year and dataset at 300‐m spatial resolution. In the case of the maps that contained multiple land cover classes (i.e., Globeland30 and Roy et al.), we selected specific classes/categories that we considered tropical dry woodlands (Appendix S1: Table S1) and reclassified the maps into a binary form (i.e., dry woodland vs. other). For converting the Global Forest Watch data into binary form, we applied a threshold of 5% woody cover at 30 m (Buchadas et al., 2022) to convert the fractional tree cover layer from 2000 into a binary woodland versus other maps. Subsequently, we masked out all areas that lost tree cover from 2001 to 2020. We left the maps already available in binary form (woodland vs. other, Reddy et al. and PALSAR) unchanged. We then aggregated all binary maps into 300‐m continuous maps by calculating the average woodland cover of 300 × 300 m pixels at 300‐m spatial resolution. Lastly, the MODIS‐TC (tree cover) and MODIS‐PNTV (non‐tree vegetation) maps were already available as continuous data (i.e., percent tree cover and percent non‐tree vegetation), and we resampled all data to 300 m using nearest‐neighbor resampling. We considered the MODIS‐PNTV product as it visually suggested high agreement with classes representing shrublands and wastelands of other classifications (e.g., Roy et al., 2015). We reprojected all data into an equal area projection for India (Lambert Conformal Conic; EPSG: 7755) and to a common extent.

**TABLE 1:** The seven datasets identified for our study include forest cover, land‐use, and land cover maps across Indian dry woodland regions.

Dataset	Period/years covered	Spatial resolution	Input data	Source
MODIS Percent Tree Cover (MODIS‐TC)	2000–2020	250 m	Satellite images	DiMiceli et al. (2015)
MODIS Percent Non‐Tree Vegetation (MODIS‐PNTV)	2000–2020	250 m	Satellite images	DiMiceli et al. (2015)
Global Forest Watch (GFW)	2000–2020	30 m	Satellite images	Hansen et al. (2013)
GlobeLand30 land cover (Globeland30)	2000/2010/2020	30 m	Satellite images	Chen et al. (2015)
PALSAR forest cover (PALSAR)	2007–2010	100 m	Satellite images	Japan Aerospace Exploration Agency (2014)
Decadal land‐use and land cover maps for India (Roy et al.)	1985/1995/2005	30 m	Satellite images	Roy et al. (2015)
Forest cover time series for India (Reddy et al.)	1880/1930/1975 1985/1995/2013	1:250,000 (1880/1930), 80 m (1975/1985); 72.5 m (1995); 56 m (2013)	Satellite images & topographic maps	Reddy et al. (2016 2017)

To evaluate the accuracy of our fractional dry woodland cover maps at 300‐m spatial resolution, we carried out an extensive accuracy assessment for each map for the latest year (2020). To do so, we first created an independent validation dataset. We randomly selected a sample of 600 grid cells across our study area with a minimum distance of 5 km between each cell. We then visually assessed each grid cell to estimate the proportion of woody cover in 10% intervals (0%–10%, 10%–20%, …, 90%–100%) using very‐high‐resolution satellite imagery from Planet and Google Earth for the focal year 2020. We used two observers to account for potential bias and combined the two assessments by taking the average of both estimations and rounding it to the nearest 5%.

**FIGURE 2:**
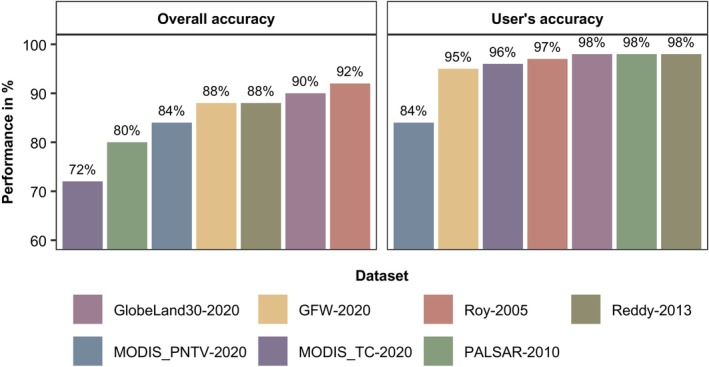
Overall accuracy and user's accuracy of seven maps depicting tree cover in Indian tropical dry woodlands for the period 2010–2020. Each of these maps was evaluated rigorously, following best practice methods as outlined by Olofsson et al. (2014).

We then compared our validation dataset with our fractional cover maps of 2020, with one map from each dataset of 2020 or the year closest. Here, we first converted our validation data, as well as the maps to be evaluated, to binary (woodlands vs. other) classes by applying different thresholds (from 15% to 22%). We then compared our validation data to these binary maps for every threshold, generated an error matrix, and calculated overall accuracies and user's accuracies of the woodland class following best practice guidelines (Olofsson et al., 2014). We recorded the threshold at which the user's accuracy of the woodland class was the highest (i.e., 20%, Figure 2). We excluded all maps from further assessments where the overall accuracy was lower than 70% and/or where the user's accuracy of the woodland class was less than 90%.

We then calculated consensus maps for the years 2010 and 2020, considering the remaining datasets by calculating the average fractional woodland cover weighted by the user's accuracy of the woodland class of the respective dataset (following the approach demonstrated in Tuanmu & Jetz, 2015):

(1) $${\mathrm{WL}}_{E}=\frac{\sum_{i=1}^{n}{\mathrm{UA}}_{i}\times {\mathrm{WL}}_{i}}{n},$$

where ${\mathrm{WL}}_{i}$ is the fractional woodland cover of map *i*, and ${\mathrm{UA}}_{i}$ is the user's accuracy of the woodland class of map *i*. We assessed the quality of our ensemble maps by comparing them to our validation points.

### Reconstructing long‐term and recent tropical dry woodland cover dynamics

To analyze dry woodland dynamics since the late 19th century, we combined our ensemble maps with a time series of historical reconstructions of woody cover in 1880, 1930, 1975, 1985, and 1995 (Reddy et al., 2017). This time series represents a combination of satellite image classifications (i.e., for the years 1975, 1985, and 1995), digitized topographic maps (i.e., 1930), and modeled woody cover (i.e., 1880) (Reddy et al., 2016) (Table 1). The maps for 1995, 1985, and 1975 were validated by Reddy et al. (2016), who used 8500 independent validation points gathered from satellite images, showing an overall accuracy of these maps ranging from 89.2% to 92.4%. The oldest maps we used, dated 1930 and 1880, were not validated in the same way due to the lack of spatially detailed validation data or high‐resolution imagery from which such validation data could be generated. However, we note that the 1930 map was derived from topographic maps (1:250,000), which can be considered of high spatial accuracy for the purpose of our analyses (at the level of 300‐m and 3‐km grid cells). The oldest (1880) map, derived through back‐casting, a spatial predictive model trained on 1930–2013 forest cover data, also underwent considerable plausibility checks and validation by Reddy et al. (2017). Specifically, this map was compared with independent estimates from Richards and Flint (1991), derived from official 1880 land records, and demonstrated a high degree of congruence. For instance, the map developed by Reddy et al. (2017) reconstructed 104.2 Mha of forest in 1880, closely aligning with the 102.7 Mha estimated by Richards and Flint (1991) for the same year. This high congruence in forest cover estimates between the two sources reinforces the plausibility of the 1880 reconstructions by Reddy et al. (2017). We applied the same homogenization strategy as in the case of our contemporary maps and aggregated the historical time series to a spatial resolution of 300 m. We considered all areas with 5% or less woodland cover during all years as non‐woodlands.

Based on our woodland cover time series, we calculated the pixel‐wise absolute percentage change for the recent years (i.e., 1995–2020) and the whole time series (i.e., 1880–2020), as well as individual decades (1995–2010, 2010–2020, etc). We considered 1995 the starting point for the recent period because it captures the beginning of important policy shifts, technological advancements, international commitments, and increased public engagement in forest conservation. We used the absolute change maps of the most recent decade (2010 to 2020) to check the plausibility of the contemporary change maps. We randomly selected 120 pixels (40 per class) from the change classes: woodland decline, stable woodland, and woodland increase. We then visually assessed whether the general trend in woodland change (e.g., loss) matched that of satellite time‐series images in Google Earth.

### Identifying major patterns of tropical dry woodland change

We summarized our woody cover time series in two ways. First, we calculated the mean and SD of all woodland pixels for each ecoregion inside our study area. Second, we used archetype analyses (Eisenack et al., 2021) to identify major patterns of woodland change. Archetype analyses are a powerful set of tools to structure and reduce complexity in social‐ecological phenomena to identify major, recurring combinations of characteristics (i.e., woodland change trajectories in our case) from a large number of heterogeneous cases (i.e., pixels in our case) (Eisenack et al., 2006; Oberlack et al., 2023). Archetype analyses have been successfully applied to identify major patterns of land‐use change (Orozco et al., 2024; Pacheco‐Romero et al., 2021) and forest change (Buchadas et al., 2022). To identify archetypes of Indian dry woodland change, we used Self‐Organizing Maps (SOMs) (Kohonen, 2001), a nonparametric clustering algorithm widely used to identify and map land system archetypes (Václavík et al., 2013). Before applying SOMs, we aggregated our 300‐m woodland cover maps to 3 km (i.e., 10 × 10 pixels) by calculating the average woodland cover. To parameterize our SOMs, we used the Kohonen package (Kohonen, 2001) in R. SOMs require pre‐setting the number of desired clusters. We tested various cluster combinations from 3 to 10 using fractional woodland cover as the input variable. We then visually evaluated each cluster based on the underlying trajectories of woodland change and spatial patterns within each cluster. Based on this assessment, we identified six clusters as the highest number possible for our data.

### Contemporary megafauna distributions and tropical dry woodland change

We compared our dry woodland cover maps and archetypes of dry woodland change with the contemporary distribution of 14 megafauna species commonly found in Indian dry woodlands. We used a smaller threshold (>20 kg; smaller thresholds have also been established for megafauna, e.g., Amir et al., 2022) for species: tiger, leopard (*Panthera pardus*), Asian elephant, chital, muntjac (*Muntiacus vaginalis*), sloth bear (*Melursus ursinus*), Indian gray wolf (*Canis lupus*), striped hyaena (*Hyaena hyaena*), dhole (*Cuon alpinus*), gaur, nilgai (*Boselaphus tragocamelus*), sambar, chinkara (*Gazella bennetti*), and blackbuck (*Antilope cervicapra*). Though occurring in dry woodland ecoregions, these species are not necessarily strictly associated with forests. While some species can be considered forest specialists (e.g., tigers), others are more generalists (e.g., elephants and leopards), and some could even be more connected to open habitats (e.g., chinkara and blackbuck). We used the range maps for these 14 megafauna species from the IUCN Red List of Threatened Species database (www.iucnredlist.org). Here, we used the range maps from the latest assessment year for each of the 14 species, ranging from 2014 to 2021. We included only extant ranges and compared each of the IUCN ranges with existing literature (Baskaran et al., 2011; Choudhury, 2002; Jhala et al., 2008; Yoganand et al., 2006) and field guides (Menon, 2014) to cross‐check whether the IUCN maps were over‐ or under‐representative of ranges. In the case of the sloth bear, we found that incorporating the “possibly extant” range aligned more closely with the current range described by Indian experts (Yoganand et al., 2006). We converted all maps into presence/absence maps at 3‐km resolution to match our woodland time series.

We conducted three analyses to explore the relationship between contemporary woodland cover (2020), long‐term woodland cover change, and our archetypes of change on the one hand, and current megafauna distributions on the other. First, we calculated Pearson's correlation coefficient between woodland cover in 2020 and the number of species present (i.e., the sum of all individual species range maps). We grouped species into two categories (threatened and non‐threatened), where threatened included all critically endangered, endangered, and vulnerable species according to the IUCN Red List (IUCN, 2012). This comprised elephant, dhole, tiger, gaur, leopard, sloth bear, and sambar. The non‐threatened group consisted of species that are near threatened and of least concern (IUCN, 2012), including nilgai, chital, chinkara, blackbuck, muntjac, Gray wolf, and Striped hyaena (IUCN, 2012). Second, to better understand the long‐term woodland cover change in present‐day species ranges, we again used the presence‐only values and plotted them against our woodland cover data for each species individually and for each year. This created a long‐term woodland cover change trajectory in the present‐day ranges of all 14 species. Third, we compared the number of species present across different archetypes, representing different archetypes of dry woodland cover change. We calculated the sum of all presence values for every pixel, grouped them based on the cluster they fell into, and calculated the median species number per cluster. We also calculated the composition of each archetype within a given species range.

## RESULTS

The ensemble map of 2020 had an overall accuracy of 90% and a user's accuracy of 97% for the woodland class, thus representing woodlands substantially better than all individual maps considered. The highest accuracy of our map was for the 20% woody cover threshold to convert our continuous woody cover maps to binary woodland/non‐woodland maps (Figure 2). We found an 80% match between the results of our absolute change map and the actual change seen in satellite images on Google Earth, suggesting our maps depict patterns of woodland change reliably.

### Long‐term and recent changes in Indian tropical dry woodlands

**FIGURE 3:**
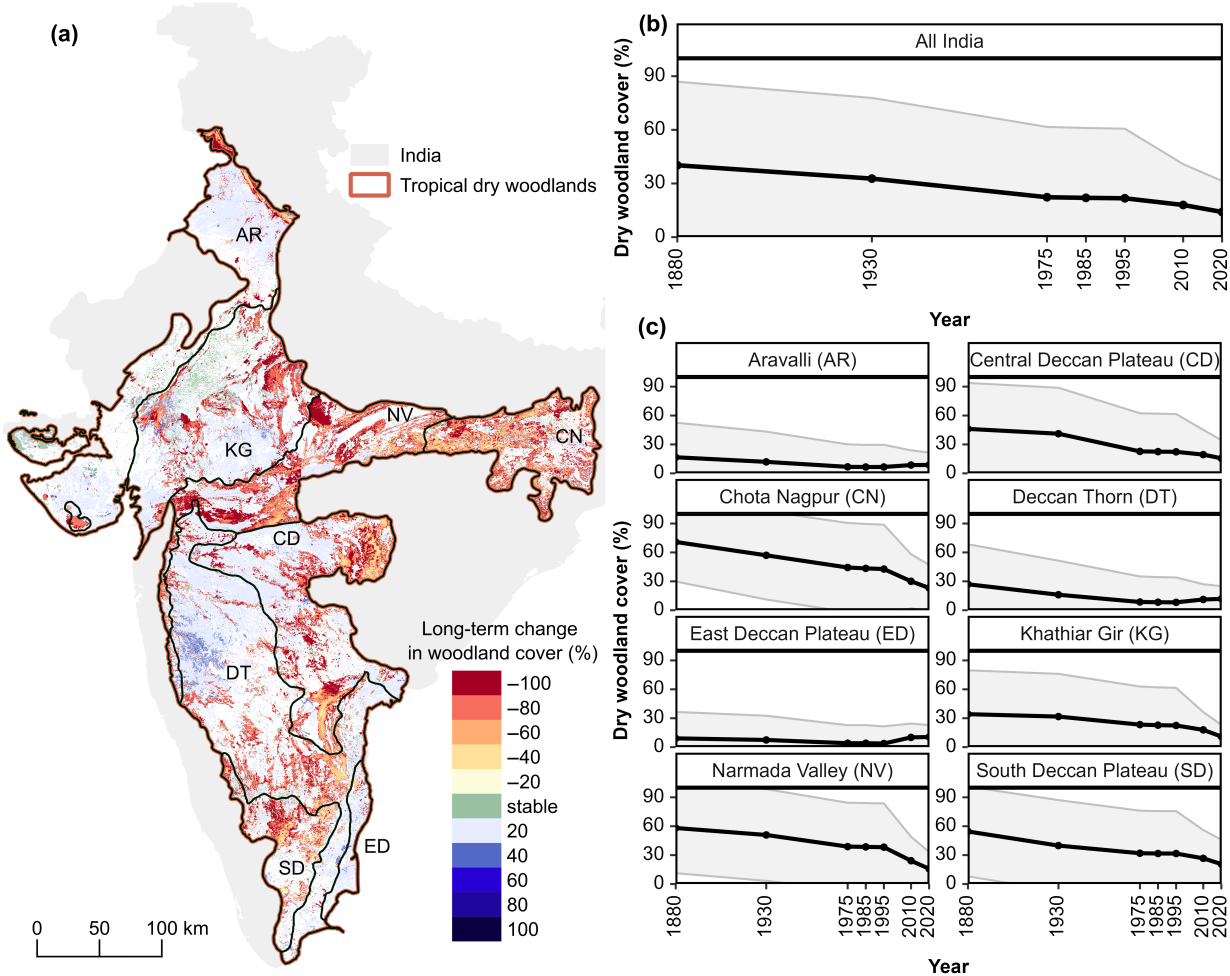
Tropical dry woodland change in India. (a) Geographical distribution of Indian tropical dry woodlands. (b) Historical changes in dry woodland cover over 140 years, represented in absolute percent change in woodland cover. (c) Historical changes in the eight dry woodland ecoregions in India, as well as for the entire country, where lines represent the trajectory of change in mean woodland cover and the shaded area indicates SD. Black circles represent the year for which we had a woodland cover map.

**FIGURE 4:**
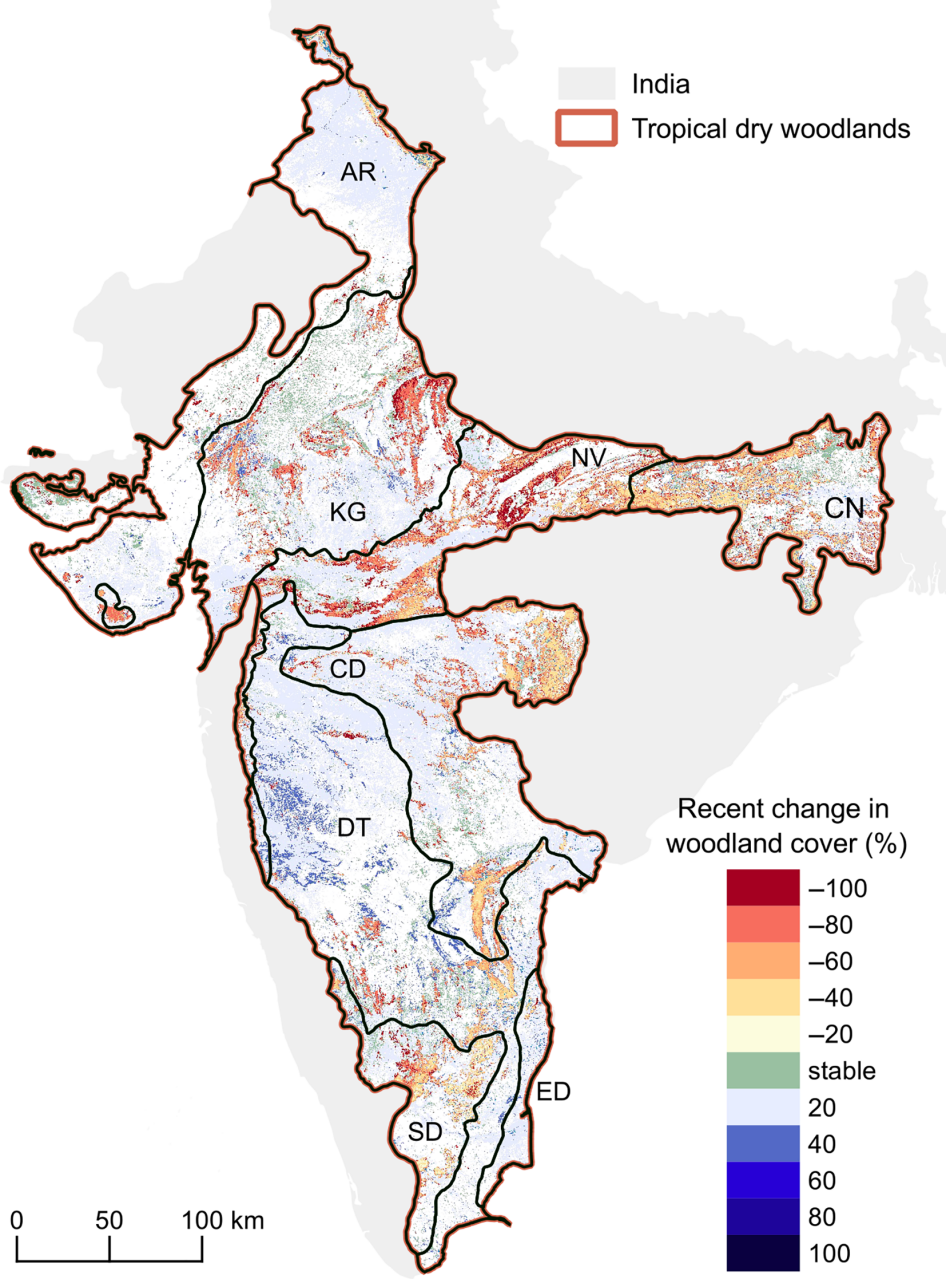
Recent (1995–2020) changes in tropical dry woodland cover in India. These results are represented as the absolute percent change in woodland cover. Here: AR = Aravalli; CD = Central Deccan Plateau; CN = Chota Nagpur; DT = Deccan Thorn; ED = East Deccan Plateau; KG = Khathiar‐Gir; NV = Narmada Valley; SD = South Deccan Plateau.

Indian tropical dry woodlands shrank substantially between 1880 and 2020, with an overall net loss in woodland cover of 22.3 Mha (Figure 3a). This resulted in a 65% loss in dry woodland area, starting at 34.4 Mha in 1880 and ending at 12.1 Mha in 2020. Overall, Indian dry woodlands were characterized by continued woodland area loss over the past 140 years (Figure 3b). More specifically, between 1880 and 1930, 6.4 Mha of tropical dry woodlands were lost, and another 8.9 Mha between 1930 and 1975. This was followed by a period of stability, between 1975 and 1995, with lower losses: 0.3 Mha (1975–1985) and 0.2 Mha (1985–1995). During recent times (1995 to 2020), over 6.5 Mha (35%) of Indian dry woodlands were lost (Figure 4). Between 2010 and 2020, Indian dry woodland extent had higher losses (3.3 Mha) than in 2000 to 2010.

Our results revealed marked geographic patterns (Figure 3a) and variation among ecoregions over the past 140 years (Figure 3c). Five ecoregions (Central Deccan Plateau, Chota Nagpur, Khathiar‐Gir, Narmada Valley, and South Deccan Plateau) had similar trajectories of gradual loss in dry woodland cover and area when aggregated to the ecoregional level across the entire historical period. The Narmada Valley experienced the highest loss in dry woodland area, losing 5.2 Mha (72.8%) over the historic period. Other noticeable losses occurred in the Central Deccan Plateau (4.7 Mha loss, 66.2%), Chota Nagpur (3.7 Mha, 67.3%), Khathiar‐Gir (3.3 Mha, 67.8%), and in the South Deccan Plateau (1.7 Mha or 62%). On the other hand, the Aravalli, Deccan Thorn, and East Deccan Plateau ecoregions witnessed a consistent loss of woodland cover and area until 1975, followed by relative stability between 1975 and 1995, and finally a gain in woodlands between 1995 and 2020. The Deccan Thorn incurred a loss of 2.8 Mha (56.4%), while Aravalli lost 0.8 Mha (47.8%). In contrast, the East Deccan Plateau was the only ecoregion to gain woodland area (0.02 Mha, 15%) over the entire historical period.

In recent times (1995 until 2020), the same five ecoregions (Central Deccan Plateau, Chota Nagpur, Khathiar‐Gir, Narmada Valley, and South Deccan Plateau) that had historical losses have continued to experience a net loss in dry woodland areas (Figure 3c). The Narmada Valley ecoregion lost the highest dry woodland area, 2.7 Mha (58.6%), followed by Khathiar‐Gir, 1.6 Mha (51%). Other notable losses occurred in Chota Nagpur (1.5 Mha, 45%), Central Deccan Plateau (1.03 Mha, 30%), and South Deccan Plateau (0.5 Mha, 34.6%). In contrast, as in the long‐term analyses, Aravalli, Deccan Thorn, and East Deccan Plateau had gains in recent times, gaining 0.2 Mha (31.7%), 0.66 Mha (43.8%), and 0.1 Mha (181%), respectively.

### Major patterns of Indian tropical dry woodland change

**FIGURE 5:**
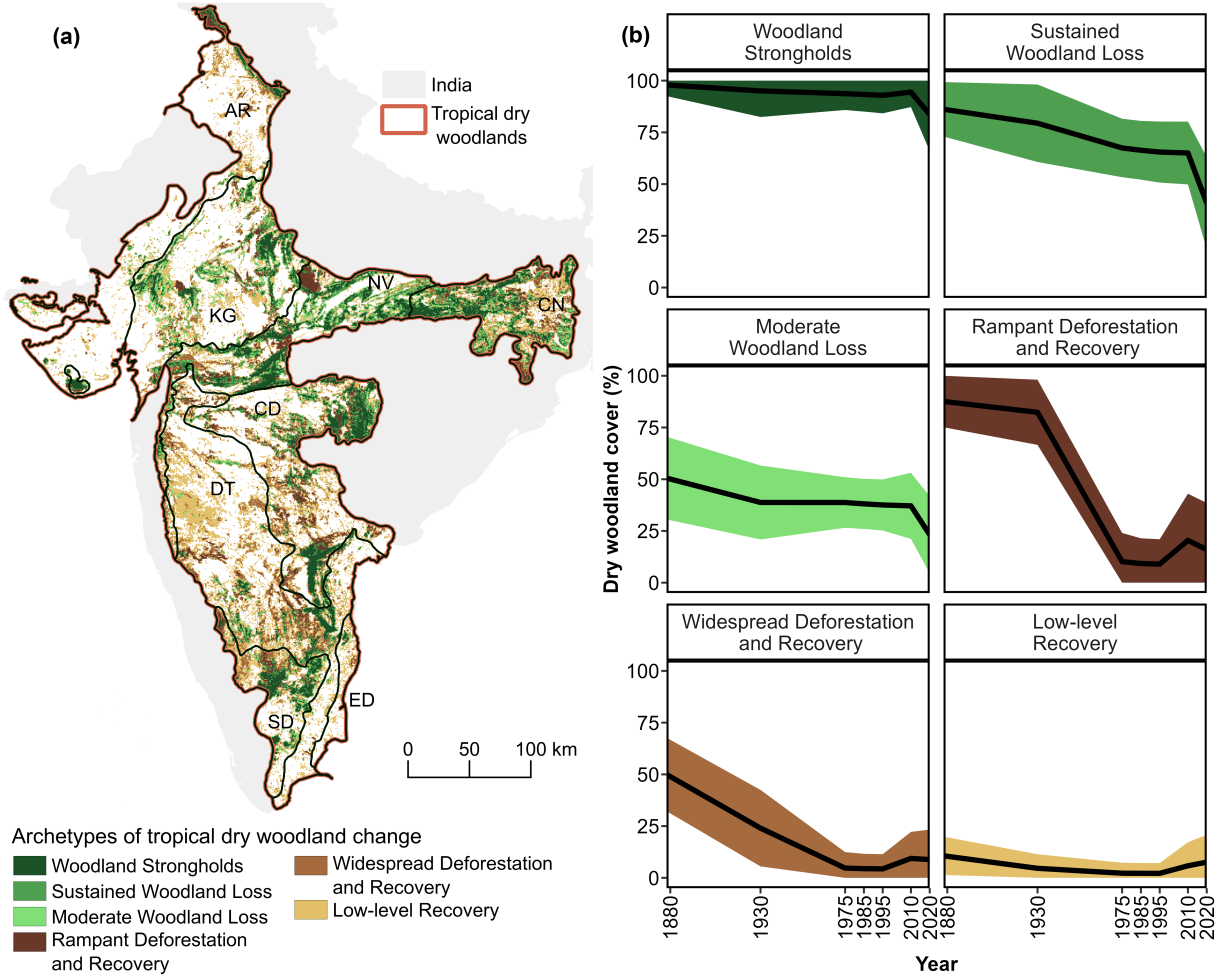
Archetypes of tropical dry woodland change in India. (a) Map of the six major archetypes identified. (b) Trajectories of tropical dry woodland cover change over 140 years for each archetype, where lines represent the average trajectory of change in woodland cover and shaded areas represent the SD.

Using our long‐term dry woodland cover time series and SOMs, we identified six distinct clusters of tropical dry woodland change in India (Figure 5a,b), varying based on (1) cluster characteristics (woodland cover, woodland area, and trajectory) and (2) geography. We interpret these clusters as archetypes of woodland change in India. Examining the trajectories of change revealed two overall similar groups of clusters: those characterized by a steady decline in dry woodland cover (archetypes: “Woodland Strongholds,” “Sustained Woodland Loss,” and “Moderate Woodland Loss”) and those clusters that exhibited a forest transition pattern of historical decline to a low point in dry woodland cover, and subsequent recovery (archetypes: “Rampant Deforestation and Recovery,” “Widespread Deforestation and Recovery,” and “Low‐level Recovery”).

Among the three archetypes characterized by the steadily declining woodland cover, the “Woodland Strongholds” archetype spanned 11.4 Mha (15.2% of the extent of tropical dry woodlands in India, Figure 5a) and was characterized by very‐high dry woodland cover across the entire 140 years we analyzed, averaging 97.9% in 1880 and 83.6% in 2020. Historically, this cluster lost 1.6 Mha (14.6%) of its woodland area, mainly in the recent (1995–2010) period, but woodland cover remained high throughout the entire time (Figure 5b). The “Sustained Woodland Loss” archetype spanned 9.1 Mha (12.1%) and was generally characterized by very‐high woodland cover, averaging 86.1% in 1880, and marked woodland loss (on average 44.4%) until 2020. Overall, this cluster lost 4.1 Mha of its woodland area, mainly before 1975, whereas woodlands were rather stable during 1975–2010, followed by a decline in the last decade. The third archetype in this group, “Moderate Woodland Loss,” had a lower average cover of 50.5% in 1880, gradually declining to an average of 23.1% in 2020, mainly in 1880–1930. After 1930, woodland cover in this archetype was fairly stable until 2010, after which it declined. This archetype covered a total area of 7.6 Mha (10.2%) and lost 2.1 Mha of its woodland area.

The three archetypes showing a forest transition pattern differed mainly in the level of initial woodland cover in 1880. The archetype “Rampant Deforestation and Recovery” was the least widespread, extending over 6 Mha (7.9%, Figure 5a). It initially had high woodland cover (on average 87.5% in 1880) but also the strongest decline, declining to 15.9% (4.2 Mha) by 2020 among all archetypes (Figure 5b). Most of the deforestation happened from 1930 to 1975, followed by stabilization (1975–1995) and partial recovery (1995–2010). Most recently, woodland cover has started to decline again. The overall pattern was similar to the archetype “Widespread Deforestation and Recovery,” spanning 11.4 Mha and characterized by a strong woodland cover decline from an average of 49.9% in 1880 to 8.8% in 2020. Historically, this archetype lost 4.7 Mha (82.3%) of its woodland area, especially before 1975, followed by periods of stability (1975–1995), brief recovery (1995–2010), and again declining woodland cover (2010–2020). Finally, the archetype “Low‐level Recovery” covered 29.4 Mha (39.1% of the total area) and was characterized by consistently low woodland cover through the study period, averaging 10.6% in 1880 and 7.5% in 2020. Historically, this archetype lost the least woodland area at 0.9 Mha (28.9%), experienced some decline until 1975, stability thereafter, and woodland recovery since 1995.

Clear geographic patterns in these six archetypes of dry woodland change in India became apparent (Figure 5a). Among individual archetypes, the “Low‐level Recovery” archetype features most prominently in the Deccan Thorn region (32%) and least in the East Deccan Plateau (2.5%). The “Widespread Deforestation and Recovery” archetype is most notable in the Deccan Thorn ecoregion (28.8%), with the least representation in the East Deccan Plateau (0.7%). The “Woodland Strongholds” archetype is found chiefly in the Narmada Valley (24.4%) and least represented in the East Deccan Plateau (0.3%). The “Sustained Woodland Loss” archetype also occurs principally in the Narmada Valley (26.2%), with the least representation in the East Deccan Plateau (0.2%). The “Moderate Woodland Loss” archetype is predominantly seen in the Khathiar‐Gir (26%) ecoregion, with the least representation in East Deccan (0.4%). Lastly, the “Rampant Deforestation and Recovery” archetype is the most noticeable in the Central Deccan Plateau (27.9%) and least in the East Deccan Plateau (0.4%).

### Indian tropical dry woodland change and contemporary megafauna distributions

**FIGURE 6:**
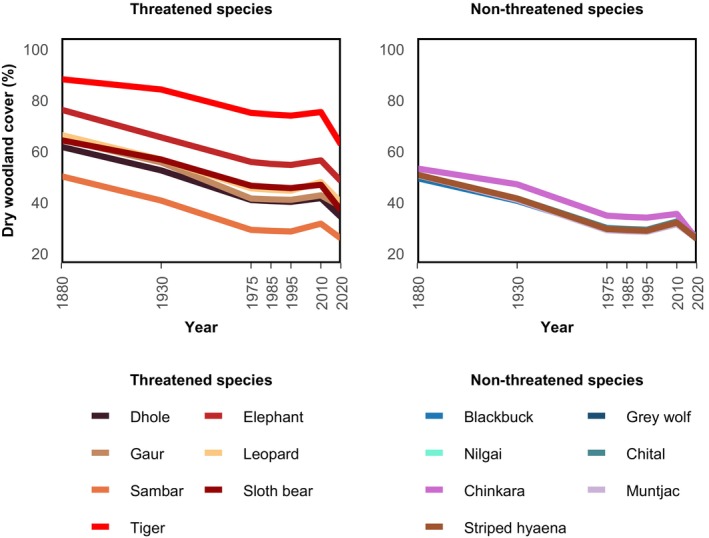
Woodland cover change in contemporary ranges of 14 megafauna species. According to the IUCN Red List of Threatened Species categories, threatened species include: critically endangered, endangered, and vulnerable. Non‐threatened species include near‐threatened and least concern.

Comparing current megafauna distribution and (1) contemporary and long‐term dry woodland cover change, as well as (2) archetypes of woodland change, revealed interesting patterns. We found a strong positive relationship between woodland cover and the number of threatened species (*r* = 0.43, *p* < 0.05). Threatened species were found in areas with 25% to 62% mean woodland cover in 2020 (Figure 6). The tiger's range had the highest dry woodland cover across time (87% in 1880 and 62% in 2020), while the sambar's had the lowest (49% in 1880 and 25% in 2020). The elephant and dhole contemporary ranges experienced the highest woodland cover loss (27.9% and 27.4%, respectively), whereas the sambar's range had the least loss (24.3%). All species' ranges showed partial woodland cover recovery between 1995 and 2010, but declined afterward.

In contrast, we found no meaningful relationship between dry woodland cover and the number of non‐threatened species, despite a significant correlation (*r* = 0.007, *p* < 0.05). Non‐threatened species occurred in areas with generally lower woodland cover in 2020 (Figure 6), with the chinkara's range having the highest woodland cover until 2010 (5.2% in 2010). There was also no clear pattern of woodland loss over time. The chinkara's range experienced the highest mean woodland cover loss (27.7%), while the blackbuck's range lost the least (23.5%).

**FIGURE 7:**
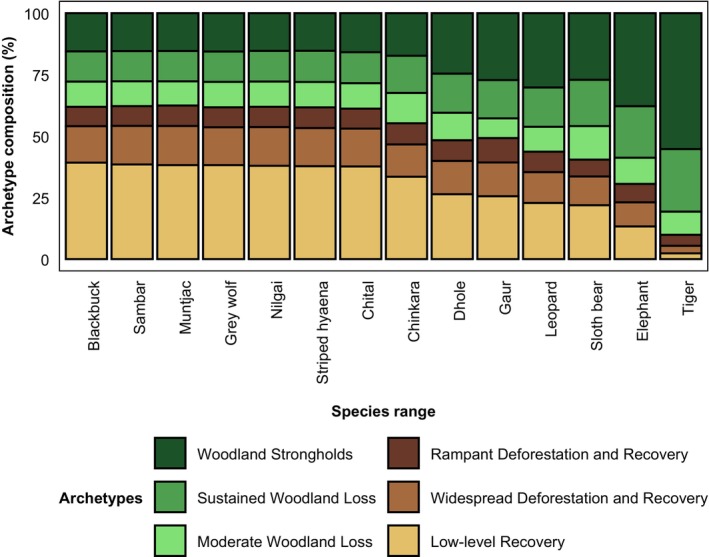
Distribution of the six tropical dry woodland change archetypes in current megafauna species' ranges.

Comparing the contemporary ranges to the six archetypes of dry woodland change showed that most archetypes had a similar number of species. Only the archetype “low‐level recovery” had fewer species according to range overlaps. Interestingly, although we analyzed the ranges of 14 megafauna species, no archetype overlapped with more than 10 species. Among the six archetypes, the “Low‐level Recovery” archetype covered over 30% of the ranges for eight species (and 26% for the dhole) (Figure 7). In contrast, the “Rampant Deforestation and Recovery” archetype was the least prevalent (7.9%–8.6%). For the remaining four species, the “Woodland Strongholds” archetype dominated their ranges (27.1%–55.2%), with other archetypes covering smaller portions.

## DISCUSSION

Tropical dry woodlands provide vital ecosystem services and support diverse wildlife, including iconic megafauna. However, they also face substantial human pressures. This is particularly true for India, where major knowledge gaps exist regarding long‐term and recent dry woodland changes. This, in turn, contributes to the prevailing bias in conservation policy and planning against dry woodlands. Here, we developed an ensemble approach using available woodland cover maps to develop a consensus map of contemporary Indian dry woodland cover and to reconstruct changes in them over 140 years. We further identified major patterns of dry woodland change and explored how these relate to the current distribution of megafauna. Our study yields four key insights. First, we highlight a massive loss of over 22 Mha (65%) of dry woodlands since 1880, underscoring the urgent need for conservation planning to safeguard India's remaining dry woodlands. Second, we identified six archetypes of dry woodland change with distinct trajectories, three showing continuous decline and three exhibiting a forest transition pattern, with recent recovery trends following historical loss. Third, we found that megafauna today primarily inhabit areas with high woodland cover, regardless of historical loss trajectories, especially for threatened species. This highlights the importance of protecting large tracts of habitats and points to megafauna recovery potential. Finally, we highlight a considerable increase in woodland loss since 1995 (6.5 Mha or 35%), pointing to high pressures on these woodlands and an urgent need to better protect them, as elsewhere in the tropics (Blackman et al., 2015; Shah et al., 2021).

Our reconstructions of tropical dry woodland change showed a 65% loss of Indian dry woodlands since the late 19th century, overall marked by three distinct phases: heavy deforestation (1880–1975), stabilization (1975–1995), and gradual loss (1995–2020). This trend broadly aligns with prior work by Richards and Flint (1991), Tian et al. (2014), and Reddy et al. (2016). However, our study extends these considerably by providing substantially enhanced temporal and spatial detail, and the generation of the first rigorously validated dry woodland cover time series. The drastic reduction of tropical dry woodlands we found through our long‐term analysis is plausible given the major pressures on dry woodlands during this time. For example, timber was especially over‐exploited, to over 200,000 ton in a single year, and supplied for military operations during World War I (Gadgil & Guha, 1992). Similarly, over 20 Mha of woodlands were converted to cropland between 1880 and 1950 (Richards & Flint, 1991), and further expansion happened during the Indian Green Revolution (1960s to 1970s), with 22 Mha converted (Tian et al., 2014). Through policies like the Indian Forest Act of 1927, the British alienated indigenous tribes who had traditionally preserved India's dry woodlands, turning these lands into open‐access resources and leading to their uncontrolled exploitation and degradation (Gadgil, 1990). In the face of these woodland losses, legislation was introduced to protect woodlands. For instance, the National Forest Policy (1952) aimed to maintain one‐third of India forested, the Wildlife (Protection) Act (1972) sought to safeguard biodiversity by establishing protected areas, and the National Forest Policy (1988) enabled a more community‐centered approach to forest management, recognizing the importance of involving local communities in conservation efforts. Together, these policies may have contributed to the stabilization of woodland cover we observed in 1975–1995.

From 1995 to 2020, we observed a continuous decline in tropical dry woodlands in India, though this decline was less drastic than in earlier periods. This trend aligns well with widely reported declines in woodland cover across India. For instance, Roy et al. (2015) reported a loss of 1.5 Mha between 1995 and 2005, Ritchie and Roser (2023) noted over 0.6‐Mha loss between 2015 and 2020, and Brandt et al. (2024) highlighted the disappearance of over 5 million trees in the woodland/farmland matrix in just four years (2018–2022). Major regional losses during the same period have also been reported from Central India (Agarwal et al., 2016) and Eastern India (Paul & Banerjee, 2021). Additionally, widespread vegetation browning and dry woodland canopy cover losses have been reported from Central India (Khanwilkar et al., 2023; Koulgi et al., 2019). Most of these losses are attributable to cropland and built‐up expansion, increased woodland dependency, and infrastructure development (Agarwal et al., 2016; Reddy et al., 2016; Roy et al., 2015).

We identified six distinct archetypical change trajectories in tropical dry woodlands in India, three of which were characterized by a steady decline in dry woodlands. Land‐use practices in India exhibit distinct regional variations driven by geographic, socioeconomic, and governance factors, leading to diverse deforestation trends, and our archetypes capture this. For instance, in western India, cropland expansion, particularly in the Narmada basin and Indira Gandhi Canal regions, is driven by mechanization and a shift toward cash crops (Roy et al., 2015; TPCG and Kalpavriksh, 2002). Southern India experiences rapid urbanization and ongoing shifting cultivation, with key biodiversity hotspots, such as the Eastern Ghats, facing forest loss due to mining and infrastructure development (Ramachandran et al., 2018; Reddy et al., 2013). In central India, deforestation and degradation are primarily linked to mining, agricultural expansion, and livestock overgrazing (Meiyappan et al., 2017; Roy et al., 2015). Our findings align with these broader geographic patterns observed across India, demonstrating region‐specific trends in dry woodland loss. This is exemplified by the “Woodland Strongholds” archetype, primarily located in the Narmada Valley, which has historically maintained high woodland cover, possibly because many woodland areas are at higher elevations, such as the Satpura and Vindhya mountains. However, recent pressures, such as large‐scale irrigation projects like the Sardar Sarovar Dam, highlight how even historically resilient woodlands are increasingly vulnerable to developmental activities (Kothari & Bhartari, 1984; Thakur et al., 2021). In contrast, the “Sustained Woodland Loss” archetype, also predominant in the region, experienced deforestation driven by colonial‐era pressures, with some stability with the enforcement of the Indian Forest Conservation Act of 1980 and the establishment of protected areas (Thakur, 2014). Similarly, the “Moderate Woodland Loss” archetype, mainly in the Khathiar‐Gir ecoregion, has seen gradual degradation and transformation of woodland habitats (Vijayan & Pati, 2002; Wikramanayake et al., 2002).

On the other hand, three archetypes point toward a transition from a historical decline in woodland cover to subsequent recovery, a pattern called forest transitions (Mather, 2007; Meyfroidt et al., 2018). Previous studies suggest that forest transitions in India occurred in the 1980s or 1990s (Mather, 2007; Singh et al., 2017), driven by five key factors: shifts in societal attitudes toward woodlands, changes in woodland product use, interactions with large‐scale systems, advancements in woodland management practices, and updates in formal evaluation and social accountability mechanisms (Bhojvaid et al., 2016). Our findings corroborate that such forest transitions occurred in dry woodlands around the mid‐1990s in some regions, such as the Deccan Thorn ecoregion, where our “Low‐level recovery” archetype showed woodland recovery post‐1995. However, we also show marked regional variation in forest transition patterns, with forest transitions happening earlier in Aravalli (1985) and somewhat later in the East Deccan plateau and Deccan thorn regions (1995), consistent with what has been found elsewhere that shows subnational trends diverging from national trends (Kuemmerle et al., 2015). This regional variation underscores the influence of localized afforestation efforts, as the number and implementation of afforestation schemes, including government programs and community initiatives, vary across different landscapes, shaping woodland recovery trajectories (Yadav & Sasaki, 2024). For instance, in the Aravalli ecoregion, internationally funded projects and Joint Forest Management initiatives have played a crucial role in restoring woodlands (Ministry of Environment and Forests and Kalpavriksh, 2004). Similarly, in South India, a combination of Joint Forest Management initiatives and agroforestry systems has contributed to woodland regeneration (Chinnamani, 1993). More generally, our results show how the spatial complexity of human‐environment interactions, different woodland change trajectories in our case, can be usefully structured through archetype analysis, helping to explain heterogeneous patterns (Rocha et al., 2020; Václavík et al., 2013).

A key finding of our study is that most threatened megafauna species are now largely confined to areas with high woodland cover. However, continued woodland loss within their ranges remains a major concern. Tigers, for instance, require woodlands for cover and prey (Johnsingh et al., 2004; Sarkar et al., 2017) but are now restricted to fragmented patches (Jhala et al., 2008). Similarly, elephants have experienced extensive habitat loss, severely impacting their populations (de Silva et al., 2023; Fernando & Leimgruber, 2011; Padalia et al., 2019). This highlights the importance of large, connected woodland patches for the long‐term survival of threatened megafauna. Conversely, adaptable species such as chital, sambar, and striped hyaena thrive across various woodland conditions from lightly to densely wooded areas (Alam & Khan, 2015; Mathur, 1991; Ramesh et al., 2013; Sankar & Goyal, 2004). Variations in land‐use practices across India drive changes in woodland cover, creating distinct archetypes that can help explain contemporary megafauna distributions. For instance, many species now inhabit areas with historically lower woodland cover, suggesting their potential to recover if land‐use pressures decline (Chapron et al., 2014; Cimatti et al., 2021; Ingeman et al., 2022). Similarly, the dominance of the “Low‐level Recovery” archetype suggests that areas undergoing gradual regeneration can support megafauna, underscoring the potential of habitat restoration efforts. However, despite numerous afforestation initiatives such as the Green India Mission, National Afforestation Program, and Joint Forest Management, comprehensive evaluations of their effectiveness in woodland recovery and their impact on megafauna remain lacking. Conversely, high woodland cover often correlates with larger protected areas, which are crucial for reducing human pressure and providing secure habitats for megafauna (Nayak et al., 2020). The prevalence of the “Woodland Strongholds” archetype highlights the critical role of intact, persistent woodlands in providing stable habitats, reinforcing the need to enhance India's protected area network to prevent further woodland loss. While the association between megafauna and woodland strongholds may seem expected, our findings underscore the importance of long‐standing woodland persistence in sustaining these populations. However, it is also plausible that megafauna persist in areas of current woodland cover regardless of historical continuity or even in non‐woodland habitats, reflecting their generalist nature and recent recovery trends in India. Regardless, our insights can inform targeted conservation strategies to effectively manage both recovering and well‐preserved woodland areas for diverse megafauna populations.

We used an ensemble approach to make the best use of a wide range of existing tree cover maps to capture long‐term and recent tropical dry woodland changes in India. While this advances our understanding of woodland dynamics and their relation to megafauna distributions, several limitations must be acknowledged. First, the oldest maps (1880 and 1930) could not be validated using satellite imagery, as the more recent maps could be, due to the general lack of historic validation data. The baseline map for 1880 was furthermore derived through a model‐based back‐casting effort, which inherently introduces uncertainty. Model reconstructions can be useful when observational data are lacking, and the 1880 map was verified using independent woodland extent statistics from official land registries (Richards & Flint, 1991). Still, although these maps depict highly plausible patterns and the trends we derive align closely with previously established timelines, we cannot fully rule out higher uncertainty associated with older maps. Similarly, our oldest maps, which are not based on satellite imagery, may be biased toward tree cover at the expense of smaller shrubs. However, our maps indicated that substantial woody cover is present in xeric shrublands and other arid areas, suggesting this bias is small. Second, the accuracy of our ensemble map still depends on the quality of the input data. While our validation suggests high accuracy, we cannot exclude remaining errors due to uncertainty in input maps. Third, while we rigorously validated our more recent input maps, our change validation relied on visual interpretation due to the absence of independent data on change. Fourth, the resolution of our woodland change maps (300 m) may miss fine‐scale details, such as small woodland patches, though it effectively captures broader patterns across India. Fifth, the difference in resolutions between our change maps (300 m) and archetype maps (3 km) may result in the cancellation of loss and gain patterns at coarser scales. While this does not affect the overall validity of the archetyping approach, the coarser resolution may obscure finer‐scale patterns. Finally, the IUCN range maps may not accurately depict species' current distributions (e.g., miss sparse occurrences outside protected areas, such as for elephants; Madhusudan et al., 2015), but this should not change the overall patterns we found.

Tropical dry woodlands have remained under‐researched, and their social‐ecological value is underappreciated by policymakers and society at large, but at the same time, dry woodlands are lost rapidly (Buchadas et al., 2023; Miles et al., 2006; Pratzer et al., 2024). This is particularly worrying because dry woodlands hold disproportionate global priority areas crucial for regulating biodiversity, carbon, and water resources (Buchadas et al., 2023). We show the same worrying trend of continuous decline for India, even in recent times. Since the early 2000s, pressures on India's dry woodlands have intensified due to a national focus on development over conservation. For instance, between 2020 and 2022 alone, the Ministry of Environment, Forest and Climate Change approved 87 development projects, including three in protected areas, potentially resulting in the removal of over 2 million trees (Jha, 2023). Given the high rates of ongoing loss and official plans to clear more dry woodlands for development (Bijoy, 2023), this highlights an urgent need for land‐use and conservation planning to safeguard India's tropical dry woodlands and their iconic, unique megafauna. Our work here shows how archetype analyses can help uncover similar trajectories of dry woodland change and their relation to megafauna distributions, which could help to devise and implement context‐specific planning.

## AUTHOR CONTRIBUTIONS

Tamanna Kalam, Tobias Kuemmerle, Matthias Baumann, and Arash Ghoddousi conceived the research idea. Parth Sarathi Roy and C. Sudhakar Reddy provided data. Tamanna Kalam and Matthias Baumann designed the analytical framework and analyzed the data with support from Florian Pötzschner and Arash Ghoddousi. All coauthors contributed to interpreting the results and writing the manuscript.

## CONFLICT OF INTEREST STATEMENT

The authors declare no conflicts of interest.

## Supporting information


Appendix S1:

## Data Availability

Data and code (Kalam et al., 2025) are available in Zenodo at https://doi.org/10.5281/zenodo.15282013.
