# Supplementary material for: Tropical dry woodland loss in India since 1880 and its relation to current megafauna distributions
Source: Ecol Appl. 2025 Jul 7;35(5):e70054. doi: 10.1002/eap.70054 (PMC12230645; doi:10.1002/eap.70054)
Supplement: Supplementary file 1 — Appendix S1: [file EAP-35-e70054-s001.pdf]

Tropical dry woodland loss in India since 1880

and its relation to current megafauna distributions

Tamanna Kalam, Matthias Baumann, Florian Pötzschner, C. Sudhakar Reddy,

Arash Ghoddousi, Parth Sarathi Roy & Tobias Kuemmerle

Journal name: Ecological Applications

Supporting Information – Appendix S1

Appendix S1: Table S1: The specific categories from the datasets below that were classified as tropical dry woodlands in our study.

<table>
<tr><th>Globeland30 land cover maps (Globeland30, Chen et al., 2015)</th><th>Decadal land use and land cover maps (Roy et al., 2015)</th></tr>
<tr><td>Forest (defined as): Lands covered with trees, with a top density of over 30%.

This includes Deciduous broadleaf forest, evergreen broadleaf, deciduous coniferous, evergreen coniferous, mixed, sparse woodland, the top density covering 10-30%.</td><td rowspan="2">1. Deciduous broad leaf forest
2. Deciduous needle leaf forest
3. Evergreen broad leaf forest
4. Evergreen needle leaf forest
5. Mixed Forest
6. Mangrove
7. Savannah/woodlands/scattered Trees</td></tr>
<tr><td>Shrubland (defined as): Lands covered with shrubs and the cover density over 30%.

This includes Mountain shrubs, deciduous and evergreen shrubs, and desert jungles in desert areas with a cover density of over 10%.</td></tr>
</table>

References

Chen, J., J. Chen, A. Liao, X. Cao, L. Chen, X. Chen, C. He, et al. 2015. "Global Land Cover Mapping at 30m Resolution: A POK-Based Operational Approach." ISPRS Journal of Photogrammetry and Remote Sensing, Global Land Cover Mapping and Monitoring 103:7–27. https://doi.org/10.1016/j.isprsjprs.2014.09.002.

Roy, P. S., A. Roy, P. K. Joshi, M. P. Kale, V. K. Srivastava, S. K. Srivastava, R. S. Dwevidi, et al. 2015. "Development of Decadal (1985–1995–2005) Land Use and Land Cover Database for India." Remote Sensing 7(3):2401–2430. https://doi.org/10.3390/rs70302401.
